# Unlocking the vital role of host cells in hair follicle reconstruction by semi-permeable capsules

**DOI:** 10.1371/journal.pone.0179279

**Published:** 2017-06-14

**Authors:** Zhexiang Fan, Yong Miao, Qian Qu, Shune Xiao, Jin Wang, Lijuan Du, Bingcheng Liu, Zhiqi Hu

**Affiliations:** Department of Plastic and Aesthetic Surgery, Nan Fang Hospital, Southern Medical University, Guangzhou, Guangdong, China; China Agricultural University, CHINA

## Abstract

Organ regeneration is becoming a promising choice for many patients; however, many details about the mechanisms underlying organ regeneration remain unknown. As regenerative organs, hair follicles offer a good model to study the mechanisms associated with regenerative medicine. The relevant studies have mainly focused on donor cells, and there are no systematic studies involving the effect of host factors on hair follicle reconstruction. Thus, we intend to explore the effect of host cells on hair follicle reconstruction. Epidermal and dermal cells from red fluorescent protein (RFP) transgenic newborn mice were injected into green fluorescent protein (GFP) transgenic mice. In addition, we wrapped the mixed dermal and epidermal cells from GFP transgenic and RFP transgenic mice by the Cell-in-a-Box kit to form "capsules," so that the cells within would be isolated from host cells. These capsules were cultured *in vitro* and transplanted *in vivo*. Fully developed reconstructed hair follicles were observed after the injection of mixed cells. These reconstructed follicles mainly consisted of donor cells, as well as a small number of host cells. The encapsulated cells gradually aggregated into cell spheres *in vitro* without apparent differentiation towards hair follicles. With respect to the transplanted capsules, concentric circle structures were observed, but no hair follicles or hair shafts formed. When the concentric circle structures were transplanted *in vivo*, mature hair follicles were observed 30 days later. Host cells were found in the reconstructed hair follicles. Thus, we conclude that host cells participate in the process of hair follicle reconstruction, and they play a vital role in the process, especially for the maturation of reconstructed hair follicles. Furthermore, we established a special hair follicle reconstruction system with the help of capsules: transplant cells were isolated from host, but other factors from host could exchange with cells inside.

## Introduction

Organ transplantation has become a good choice for people whose organs are lost or damaged as a result of disease, injury, or aging [1,2]; however, there are still many challenges, in which inadequate supply of organs and immunologic rejection constitute the main parts [3,4]. With the recent development of regenerative medicine and tissue engineering, organ regenerative therapy has become a promising solution to the inadequate supply of organs and immunologic rejection [5]; however, current technology is not sufficient to allow the reconstructed tissue to effectively mature and dynamically adjust to the new environment after transplantation [6]. Hair follicles are regenerative mini-organs that periodically and stereotypically regenerate throughout life, thus hair follicles constitute an important model for organ regeneration [7,8]. Indeed, we can successfully reconstruct mature hair follicles using numerous *in vivo* animal models, such as the chamber assay, patch assay, flap assay, and sandwiches [9–13]. Although these methods have implemented the cross between organs and scattered cells, such methods are only suitable for detecting the hair-inducing capacity of cells. In-depth knowledge of hair follicle reconstruction is easier to acquire, which may help better elucidate the mechanisms underlying regeneration in other organs. *In vivo* models are inapplicable for analyzing single factors due to many factors involved, while *in vitro* experiments can solve the problem effectively. Nevertheless, at present we can only form hair follicle-like structures *in vitro*, which need to be adopted *in vivo* for further maturity [14]. Thus, the microenvironment is not suitable for hair follicle reconstruction *in vitro* at present; however, few reports have explored whether or not there is a lack of specific humoral or cellular factors that contribute to such inefficiency. Cells used in *in vitro* and *in vivo* hair reconstruction models are the same. In the current study, we sought to explore whether or not host cells participate in the process of hair follicle regeneration directly when injected under the panniculus carnosus. With the aid of isolation technology of transplanted cells, we explored the influence of host cell factors on hair follicle reconstruction *in vivo*, and determined whether or not completely developed hair follicles can form without the participation of host cells. This research provided some useful experience and will provide guidance for constructing other organs. The underlying mechanism of hair follicle reconstruction remains the target of ongoing research.

## Materials and methods

### Animals

Ten newborn (0 days old) C57BL/6J mice and 18 nude male mice (Balb/c, nu/nu; 4–6 weeks old) were obtained from the Experimental Animal Centre of Southern Medical University (Guangzhou, China). Sixteen newborn (0 days old) red fluorescent protein (RFP) mice were obtained from Beijing Vitalstar Biotechnology (Beijing, China). Ten newborn (0 days old) and 24 adult (6–8 weeks old) female green fluorescent protein (GFP) mice were obtained from the Institute of Animal Models of Nanjing University (Nanjing, China). All animal experiments were performed under the approval of the Southern Medical University Animal Care and Use Committee.

### Preparation of cells for *in vivo* grafting

Full thicknesses of dorsal skin were derived from newborn RFP mice at natal day 0. The dermis and epidermis were separated using dispase (Sigma, St. Louis, MO, USA) by incubation at 4°C overnight. The piece of skin was rinsed three times with phosphate-buffered saline (PBS, Gibco, Grand Island, NY, USA), then the skin piece was split into epidermis and dermis with forceps. Each component was minced. The dermis was digested in 0.2% collagenase (Sigma, St. Louis, MO, USA) at 37°C for 1 h. After digestion, an equal volume of Dulbecco’s modified Eagle’s medium (DMEM, Gibco, Grand Island, NY, USA) supplemented with 10% fetal bovine serum (FBS, Gibco, Grand Island, NY, USA) was added, and the cell suspension was filtered sequentially through 100 μm and 40 μm mesh cell strainers. The cell suspension was centrifuged at 230 g for 5 min, then the cell pellet was resuspended in DMEM. The epidermis was digested in 0.25% trypsin-EDTA (Gibco, Grand Island, NY, USA) at 37°C for 10 min to obtain freshly isolated epidermal cells, as previously reported [15]. The preparation of cells from GFP newborn mice is the same as previously described.

### Preparation of capsules

Ninety milliters of water was pipetted into a 250 ml beaker, then 10 ml of solution 1from Cell-in-a-Box kit (Sigma, St. Louis, MO, USA) was added. The pipette was rinsed in the hardening bath. The mixture was stirred for 10 min. For encapsulation, the speed of the stir bar was reduced to the lowest practical speed. The cells were washed twice in PBS and counted. Dermal cells (1.4×10^6^) from GFP newborn mice and epidermal cells (0.7×10^6^) from RFP mice were placed in a sterile 1.5 ml microcentrifuge tube. The cells were centrifuged at 200g for 5 min and the supernatant was discarded. One milliliter of solution 1 was added to the cell pellet and the pellet was resuspended by pipetting up and down until the cells were uniformly dispersed. The formation of air bubbles was avoided. A red plastic filling needle (G18½, blunt end) was added to a 1 ml Luer lock syringe and the cell suspension was drawn up. The filling needle was replaced with a green plastic droplet needle (G34, blunt end), taking especial care to assure that the needle was screwed firmly in place. Air bubbles were eliminated from the syringe. The needle was held vertically, 2–3 cm above the hardening bath. Droplets were dispensed at a moderate rate of 1–2 drops per second while maintaining the same drop height. The needle was moved around slightly to prevent droplets from landing at the same spot in the bath. We continued to make as many capsules as required, but did not dispense droplets after 1 min. After dispensing the last droplet, the capsules were stirred for 5 min. The stirrer speed was adjusted to ensure that the capsules were moving continuously in the bath. The stirrer was stopped and the capsules were allowed to settle. Fifty milliliters of the bath solution were discarded using a serological pipette, then 100 ml of sterile PBS was dispensed into the beaker. Stirring was restarted to wash the capsules for 10 min. One hundred milliliters of the bath solution was discarded and 100 ml of fresh sterile PBS was added into the beaker. The bath solution was washed again for 5 min. The remaining PBS was discarded, leaving just enough liquid to cover all of the capsules. The bath solution was washed three times with 30 ml of PBS, then three additional times with 30 ml of cell culture medium. A rinse cycle was performed by the addition of 30 ml of liquid, which was then removed by pipette. The preparation of blank capsules was the same as above.

The capsules were picked up with a 25 ml serologic pipette, and placed in culture dishes. An appropriate volume of cell culture medium was added (a mixture of DMEM containing 10% FBS and keratinocyte serum-free medium (K-SFM, Sigma, St. Louis, MO, USA) at a ratio of 2:1). Then, the dishes were incubated at 37°C and 5% CO_2_ in air. The medium was changed at regular intervals 2–3 times a week. The capsules were observed regularly. Cell spheres were removed from some capsules 20 days later, which were cultured *in vitro*. Some of the cell spheres were reseeded in culture dishes and the migration of these cells was observed under a microscope. Other cell spheres were harvested for paraffin sectioning and hematoxylin-eosin (HE) staining. The remaining capsules were cultured for an additional 10 days.

### Transplantation of capsules and cells

GFP and nude mice were cleaned with Betadine and put under anesthesia by intraperitoneal injection of 10 g/l pentobarbital sodium (0.4 ml/100 g). The capsules that contain dermal cells from GFP newborn mice and epidermal cells from RFP newborn mice were then transplanted subcutaneously into nude mice. The blank capsules were also transplanted as the control group. Each group contained six mice and each mouse had one site where 10 capsules were transplanted as a whole. Cell mixtures from RFP mice (dermal cells, 5×10^6^; epidermal cells, 5×10^6^) in a total volume of 100 μl, were injected under the panniculus carnosus of the GFP mouse skin. Each mouse was injected at six points. In addition, dermal and epidermal cells were also injected as a control. Each group contained 6 mice as well. After recovery from anesthesia, mice were caged individually.

### Observation and evaluation of hair follicle reconstruction

Skin color changes at the injected site were monitored every day. Fourteen days later, the mice were killed by an overdose injection of anesthetic, the skin of the injected site was harvested and the sample was observed with stereoscopic microscopy. Paraffin sections were prepared and the histologic sections were stained with HE. Frozen sections and 4′,6-diamidino-2-phenylindole (DAPI, Sigma, St. Louis, MO, USA) staining were made. The specific distribution of the fluorescent cells in the reconstructed hair follicle was observed in the frozen sections. Some of the graft sites where the capsules were transplanted were harvested 10 d later, and observed under a stereoscope and upright microscope. Cell spheres were removed, some of which were harvested for paraffin sections and HE staining, others were transplanted into nude mice. The hair follicle formation was monitored. Other capsules were retained *in vivo* and were harvested and assessed 40 d later.

The above steps were repeated with dermis and epidermis cells from newborn RFP mice. Capsules were transplanted into GFP mice. Ten days after transplantation, cell spheres inside the capsules were harvested, then transplanted into GFP mice. Similarly, the hair follicle reconstruction was observed.

## Results

### Host cells participated in the hair follicle regeneration process

**Fig 1:**
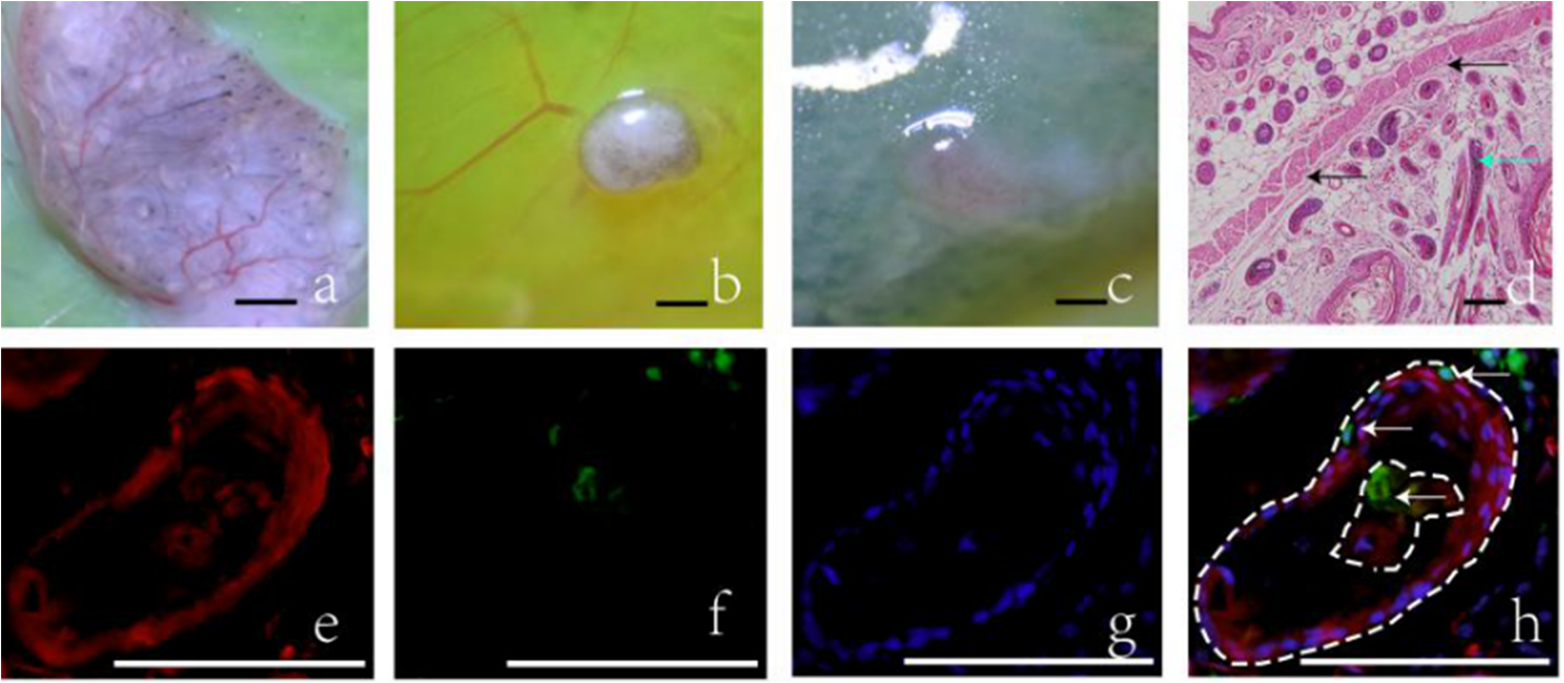
The hair regeneration of cells from RFP mice when injected into GFP mice. (a) Abundant hair follicles were reconstructed when dermal and epidermal cells were injected together, but no hair follicles were reconstructed when epidermal cells (b) or dermal cells (c) were injected alone. (d) Fourteen days after the injection of dermal and epidermal cells: HE sections, arrows indicate the regenerated HFs (green) and the panniculus carnosus (black). (e, f) Frozen sections of reconstructed hair follicles observed under a fluorescence microscope, red indicates donor cells, green indicates host cells. (g) DAPI staining of reconstructed hair follicle. (h) Synthesis, arrows indicate the green fluorescent cells (white). Scale bars = 1 mm in a, b, and c; 100 μm in d, e, f, g, and h.

To certify whether or not the host cells were involved in the hair follicle regeneration process, we transplanted combined cell mixtures, as well as single cells, into mice to form hair follicles. Dermal and epidermal cells from RFP mice were injected into GFP mice at a ratio of 2:1, and blebs were observed. The injected site became gray 4 d later and turned black 8 d later. Fourteen days later, the injected sites were harvested for detection. Abundant hair follicles were observed in sites injected with mixed cells (Fig 1A); however, when injected alone, epidermal and dermal cells failed to form hair follicles (Fig 1B and 1C). The completely developed structure of hair follicles was observed under the panniculus carnosus, and the panniculus carnosus was continuous and complete (Fig 1D). Frozen sections of the grafts were prepared and examined under a fluorescence microscope. The results indicated that newly formed follicles were mostly composed of red fluorescent cells, as well as a small number of green fluorescent cells (Fig 1E–1H), conclusively demonstrating that the host cells were involved in the process of hair follicle regeneration.

### Environment created by capsules is suitable for cell growth

**Fig 2:**
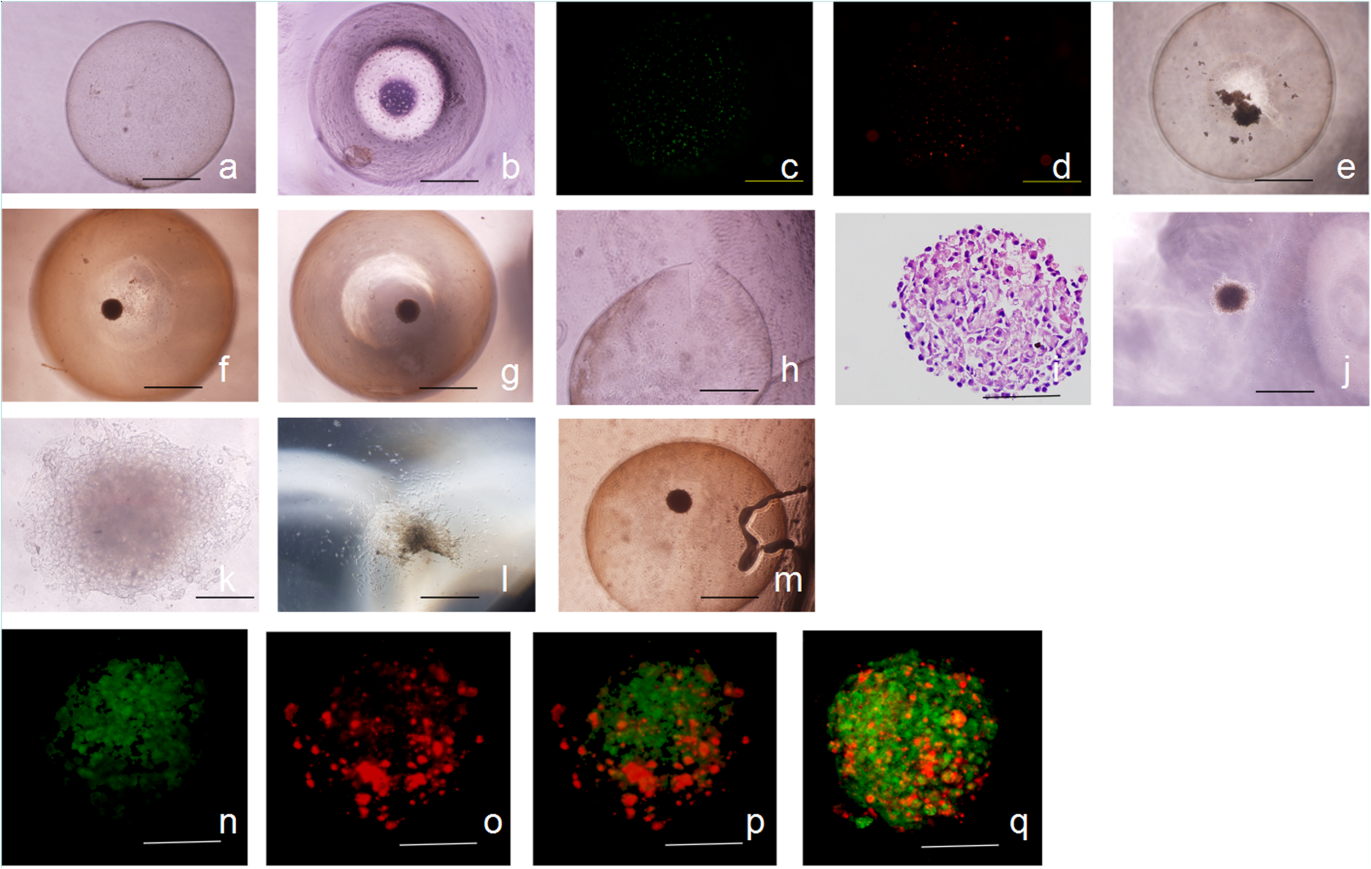
The cultivation of capsules *in vitro*. Dermal and epidermal cells were encapsulated in capsules (a). Cells in the capsules gradually aggregated into small multicellular aggregates (b). Dermal cells (red) and epidermal cells (green) were observed under an inverted fluorescence microscope (c, d). Four days later, multicellular aggregates merged into hybrid spheroids (e). Seven days later, the morphology of hybrid spheroids became stable (f, g [10 d after encapsulation]). Twenty days after capsules were cultured *in vitro*, cell spheroids were taken out of capsules (h). HE sections of cell spheres were made; neither hair follicles nor concentric circles were observed (i). Cell spheroids attached to the wall when reseeded in culture dishes; cells inside then migrated from the spheroids (j–l, 0 d, 2 d, and 7 d after reseeding, respectively). The capsules were cultured *in vitro* for 30 d, no apparent change was observed (m). Confocal micrographs taken at 7d (n–p). These images showed dermal (green) and epidermal cells (red) in cell spheroids. The z reconstituted image clearly showed that dermal cells were located in the center and epidermal cells were sorted to the surface (q). Scale bars = 1 mm in a, b, c, d, e, f, g, h, j, l, and m; 200 μm in k; 100 μm in i, n, o, p, and q.

To verify the function of the involved host cells in the process of hair follicle reconstruction, fresh epidermal cells from RFP newborn mice and dermal cells from GFP newborn mice were encapsulated with the Cell-in-a-Box kit. We generated approximately 100 capsules every time, each capsule contained 2.1×10^4^ cells. Capsule diameter was 3.13±0.16 mm (mean±standard deviation). With the use of capsules, we isolated the injected cell mixtures from the host cells. We found that cells in the capsules gradually aggregated into small multicellular aggregates *in vitro* (Fig 2A and 2B), which are highly motile. Dermal and epidermal cells came from different fluorescent protein transgenic mice, they were observed under an inverted fluorescence microscope (Fig 2C and 2D). Four days later, multicellular aggregates aggregated into a hybrid spheroid (Fig 2E). Seven days later, the morphology of hybrid spheroids became stable, spheroids were almost uniform in size, the diameter was 281±7.5 um (Fig 2F), but there were no significant changes in the subsequent culture (Fig 2G). Twenty days later, cell spheroids were taken out of some capsules after *in vitro* cultivation (Fig 2H); no apparent differentiation was observed through the HE section of hybrid spheroids (Fig 2I). To eliminate this possibility that the lack of nutrients resulted in the death of encapsulated cells, some cell spheroids were then reseeded in culture dishes with the medium (DMEM containing 10% FBS and K-SFM [mixed 2:1]). The spheroids then attached to the dishes and the cells migrated from the spheroids (Fig 2J–2L). The remaining capsules were cultured *in vitro* for another 10 d, but no apparent change was observed (Fig 2M). With the help of laser confocal microscopy, we discovered that dermal cells were preferentially located in the center and epidermal cells were sorted to the surface (Fig 2N–2Q). The spontaneously formed layered structure of hybrid spheroids was similar to the natural three-dimensional organization of the hair bulb (a shell of keratinocytes surrounding the center of aggregated dermal papilla cells). The above results showed that the environment in capsules can support the survival of cells. The epidermal and dermal cells in the capsules aggregated gradually, which is similar to the state in the three-dimensional environment created by the hanging drop culture or the matrigel [16–18]. Nevertheless, the hybrid spheroid appeared to be the final outcome, and failed to continue the differentiation toward the structure of the hair follicle.

### The host cells are necessary for the maturation of reconstructed hair follicles

**Fig 3:**
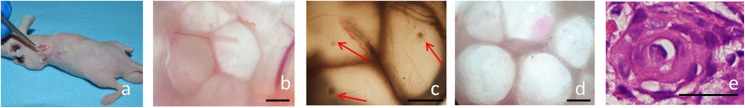
Capsules transplanted *in vivo*. (a) Capsules were taken out. (b–d) Observed under the microscope, arrows indicate cell spheroids (red). (e) Thirty days after the capsules were transplanted subcutaneously into nude mice, HE sections, and cells formed concentric circles. Scale bars = 1 mm in b, c, and d; 50 μm in e.

**Fig 4:**
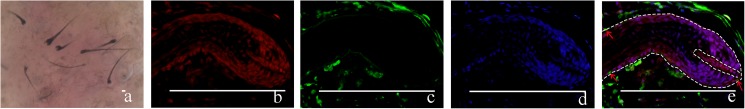
Hair reconstruction of the cell spheroids inside the capsules 10 d after the transplantation. (a) Cell spheres inside the capsules were acquired for single transplantation into nude mice. Thirty days after transplantation, mature hair follicles were reconstructed successfully. (b) Red fluorescence of the reconstructed hair follicle. (c) Green fluorescence figure of the reconstructed hair follicle. (d) DAPI staining of the reconstructed hair follicle. (e) Synthesis, arrows indicate GFP cells from host mice (red). Scale bars = 50 μm in a; and 100 μm in b, c, d, and e.

Whether or not the lack of some growth factors or host cells lead to the failure of cell mixtures developing into hair follicles *in vitro* is unknown. To explore the possibility that cells in capsules could reconstruct hair follicles *in vivo*, we used capsules and the cells taken from the capsules for transplantation. Intact capsules, which contain epidermal and dermal cells, were transplanted into nude mice. No adverse effects were observed following capsule transplantation. Such being the case, we mimicked the microenvironment *in vivo*; transplanted cells could accept all the nutrition from the host, but were also insulated from host cells. Blank capsules were transplanted as a control. The wound healed well. Ten days later, some transplanted sites were harvested (Fig 3A). Transplanted capsules were intact, inside of which the cells formed spheroids, and similar to *in vitro* cultures (Fig 3B and 3C). There was nothing in the blank capsules (Fig 3D). HE sections indicated that the cells formed concentric circles, whereas no hair follicles or obvious hair shafts were found (Fig 3E). Thus, cells in capsules differentiated toward hair follicles *in vivo*. To verify whether or not the immature hair follicle-like structures could mature, the other transplanted sites were harvested 40 d later, yet there were still concentric circles without any mature follicles. In conclusion, even if supplied with sufficient nutrients from the host, the encapsulated cells could not form mature hair follicles when insulated from host cells. Other cell spheroids harvested 10 d after *in vivo* transplantation were transplanted into nude mice. As expected, 30 d after transplantation, approximately 70%–80% of spheroids developed mature hair follicles. We then repeated the above steps with dermal and epidermal cells from RFP mice, and capsules were transplanted into GFP mice. Ten days later, cell spheres were harvested and transplanted into GFP mice. Mature hair follicles were also observed (Fig 4A), approximately 70%–80% of spheroids developed mature hair follicles. New reconstructed follicles were mostly constituted of red fluorescent cells, as well as a small number of green fluorescent cells (Fig 4B–4E).

## Discussion

Currently, the replacement of dysfunctional or missing organs in a recipient by a healthy and fully functioning donor organ is an essential treatment, which is widely applicable to various organs, such as the lung, liver, pancreas, and heart [19,20]. Developing regenerative medicine holds the promise of tissue and organ regeneration; however, many details about the mechanisms underlying organ development, regeneration, and healing remain unknown. As regenerative organs, hair follicles offer a highly informative model to study the mechanisms of systems biology and regenerative medicine. In this article, we chose to study the hair follicle, the morphogenesis of which depends on the complex epithelial-mesenchymal interactions, as a tool to research the mechanism underlying organ regeneration [21,22]. Various studies have confirmed that hair follicles can be reconstructed by transplanting appropriate cells *in vivo* [9–13], but the mechanism behind the process is not fully clear. Nevertheless, at present we cannot reconstruct mature hair follicles *in vitro*, and there is no systematic research on the effect of host factors on hair follicle reconstruction [23].

It is well-known that the microenvironment plays an important role in cell proliferation, differentiation, and function maintenance [24]. The microenvironment of the host also exercises a great influence on the reconstruction of hair follicles [25]. The microenvironment typically consists of the following sections: cells (such as white blood cells, fibroblasts, and adipocytes), growth factors (such as transforming growth factor, vascular endothelial growth factor, and fibroblast growth factor), the extracellular matrix (laminin, fibronectin, and proteoglycan), and signaling molecules (WNT, bone morphogenetic protein, and Notch) [26–28]. Whether or not the difference in the microenvironment between *in vivo* and *in vitro* models plays an important role in hair follicle regeneration is unknown. Thus, we investigated the influence of host cells in hair follicle regeneration first.

To certify whether or not the host cells involved in hair follicle regeneration process, dermal and epidermal cells from RFP mice were injected under the panniculus carnosus of GFP mice. Abundant hair follicles were observed only when dermal and epidermal cells were injected together. By utilizing frozen sections, we observed that these reconstructed follicles mostly consisted of red fluorescent cells, as well as a small number of green fluorescent cells, which were derived from the host. We thus concluded that the host cells were involved in the process of hair follicle regeneration *in vivo*. This experiment was the first use of the spontaneous fluorescence cells in the research about host cells in the hair follicle reconstruction process. The operation is much more simple and credible, and not only avoids immunohistochemical staining to trace the transplanted cells, but also avoids the error caused by the use of fluorescent dyes, eliminating the injury of fluorescent dye on cells [29,30].

The function of these host cells in the process of hair follicle reconstruction remains unknown. To this end, we are committed to looking for a system which can isolate the host cells and does not affect the exchange of signaling interflow between the host and transplanted cells.

The Cell-in-a-Box technology from Austrianova enables one to encapsulate cells in a protective, semi-permeable, cellulose-based bead. Small pores in the beads allow for the exchange of the nutrient and waste, but retain the cells within the beads. The beads are durable; capable of withstanding up to 6 months in an implant. Furthermore, the beads are well-tolerated, so a host immune response will not be elicited. These characteristics allow the embedded cells to grow for longer periods than traditional two-dimensional cell culture. This technology has already been used successfully in novel research and clinical applications [31,32]. Therefore, we chose the Cell-in-the-Box as the method for unique analyses of the interaction between the host environments and transplanted cells.

We confirmed that spontaneous flourescence epidermal and dermal cells were encapsulated in capsules. We also observed the spatial distribution of different cells. With the help of laser confocal microscopy, we discovered that dermal cells were preferentially located in the center and epidermal cells were sorted to the surface, which is similar to the results of previous research [33]. We demonstrated that the environment in capsules is suitable for the survival of cells. But the *in vitro* environment in our research cannot support the differentiation of cells towards hair follicles. When capsules were transplanted *in vivo*, due to the characteristics of capsules, cells in capsules were supplied with sufficient nutrients from hosts. Concentric circles were observed, which indicated that cells in capsules differentiated toward hair follicles. It indicated that body fluid plays an important role in the differentiation of hair follicles. Because the hair follicle-like structures could not form mature hair follicles when insulated from host cells, we then took the spheroids out of the capsules 10 d after *in vivo* transplantation, and transplanted the spheroids alone into nude mice. As expected, hair follicles reached maturation. To verify whether or not host cells exist in the reconstructed hair follicles under such circumstance, we chose dermal and epidermal cells from RFP mice as the donor cells and GFP mice as the host, then repeated the above steps. In like manner, mature hair follicles formed successfully. Furthermore, the new reconstructed follicles mostly constituted of red fluorescent cells as well as a small number of green fluorescent cells. Eventually, we concluded that host cells play a vital role in the process of hair follicle reconstruction. With normal microenvironment, host cells play a vital role in the process of morphogenesis and maturation of hair follicles, good external signal environment under the premise of normal host cells plays a vital role.

From my prospective, there are two key points may illustrate the significance of the host cells in the reconstruction of hair follicle. Firstly, the host cells may constitute the necessary components of tissue, which could not be replaced with cells transplanted. Secondly, the host cells are major compartments of the microenvironment in vivo. They can contact with the transplanted cells and supply special signal molecules, promoting organ morphogenesis and maturation. The interactive relationship between the host cells and transplanted cells exert a pivotal role in the process of hair follicle reconstruction [34,35].

The hair follicle is an accessible and clinically relevant organ regeneration model. It has been applied widely for organ regeneration research [8]. This research could provide some experience about hair follicle reconstruction and some other organs. However, hair follicles are miniature ectodermal organs, so they could not fully represent mesoblastic and hypoblastic complex organs, such as liver, lung, muscle, bone and so on. More research need to be done.

## Conclusions

In summary, our study is the first to systematically focus on the details underlying the reconstruction of hair follicles from the aspect of host factors. With the help of spontaneous fluorescence cells and the Cell-in-a-Box technology, we confirmed that host cells were involved in the process of reconstruction of hair follicles. In addition, we further demonstrated that these host cells are necessary for the maturation of reconstructed hair follicles. Based on the experiments, we know more details of hair follicle regeneration, which provides a new direction for the reconstruction of other organs. Yet, more research about the mechanism of hair follicle reconstruction is needed.

## References

[1] Ellis JA, Sinclair RD. Male pattern baldness: current treatments, future prospects. Drug Discov Today. 2008;13: 791–797. doi: 10.1016/j.drudis.2008.05.010 18617016 10.1016/j.drudis.2008.05.010

[2] Ikeda E, Morita R, Nakao K, Ishida K, Nakamura T, Takano-Yamamoto T, et al Fully functional bioengineered tooth replacement as an organ replacement therapy. Proc Natl Acad Sci U S A. 2009;106: 13475–13480. doi: 10.1073/pnas.0902944106 19666587 10.1073/pnas.0902944106 PMC2720406

[3] Orlando G, Soker S, Stratta RJ. Organ bioengineering and regeneration as the new Holy Grail for organ transplantation. Ann Surg. 2013;258: 221–232. doi: 10.1097/SLA.0b013e31829c79cf 23782908 10.1097/SLA.0b013e31829c79cf

[4] Bolton EM, Bradley JA. Avoiding immunological rejection in regenerative medicine. Regen Med. 2015;10: 287–304. doi: 10.2217/rme.15.11 25933238 10.2217/rme.15.11

[5] Mao AS, Mooney DJ. Regenerative medicine: current therapies and future directions. Proc Natl Acad Sci U S A. 2015;112: 14452–14459. doi: 10.1073/pnas.1508520112 26598661 10.1073/pnas.1508520112 PMC4664309

[6] Asakawa K, Toyoshima KE, Ishibashi N, Tobe H, Iwadate A, Kanayama T, et al Hair organ regeneration via the bioengineered hair follicular unit transplantation. Sci Rep. 2012;2: 424 doi: 10.1038/srep00424 22645640 10.1038/srep00424 PMC3361021

[7] Toyoshima KE, Asakawa K, Ishibashi N, Toki H, Ogawa M, Hasegawa T, et al Fully functional hair follicle regeneration through the rearrangement of stem cells and their niches. Nat Commun. 2012;3:784 doi: 10.1038/ncomms1784 22510689 10.1038/ncomms1784 PMC3337983

[8] Yu BD, Mukhopadhyay A, Wong C. Skin and hair: models for exploring organ regeneration. Hum Mol Genet. 2008;17(R1):R54–9. doi: 10.1093/hmg/ddn086 18632698 10.1093/hmg/ddn086

[9] Xiao S, Hu Z, Jiang J, Miao Y, Feng C. Neonatal murine skin-derived cells transplanted using a mini-chamber model produce robust and normal hair. J Tissue Eng Regen Med. 2016;10: E286–293. doi: 10.1002/term.1802 23950039 10.1002/term.1802

[10] Lichti U, Anders J, Yuspa SH. Isolation and short-term culture of primary keratinocytes, hair follicle populations and dermal cells from newborn mice and keratinocytes from adult mice for in vitro analysis and for grafting to immunodeficient mice. Nat Protoc. 2008;3: 799–810. doi: 10.1038/nprot.2008.50 18451788 10.1038/nprot.2008.50 PMC6299324

[11] Zheng Y, Du X, Wang W, Boucher M, Parimoo S, Stenn K. Organogenesis from dissociated cells: generation of mature cycling hair follicles from skin-derived cells. J Invest Dermatol. 2005;124: 867–876. doi: 10.1111/j.0022-202X.2005.23716.x 15854024 10.1111/j.0022-202X.2005.23716.x

[12] Su YS, Miao Y, Jiang JD, Liu H, Hu J, Hu ZQ. A simple and rapid model for hair-follicle regeneration in the nude mouse. Clin Exp Dermatol. 2015;40: 653–658. doi: 10.1111/ced.12563 25623661 10.1111/ced.12563

[13] Qiao J, Philips E, Teumer J. A graft model for hair development. Exp Dermatol. 2008;17: 512–518. doi: 10.1111/j.1600-0625.2007.00661.x 18070082 10.1111/j.1600-0625.2007.00661.x

[14] Qiao J, Turetsky A, Kemp P, Teumer J. Hair morphogenesis in vitro: formation of hair structures suitable for implantation. Regen Med. 2008;3: 683–692. doi: 10.2217/17460751.3.5.683 18729793 10.2217/17460751.3.5.683

[15] Woo WM, Atwood SX, Zhen HH, Oro AE. Rapid genetic analysis of epithelial-mesenchymal signaling during hair regeneration. J Vis Exp. 2013: e4344 doi: 10.3791/4344 23486463 10.3791/4344 PMC3622109

[16] Higgins CA, Chen JC, Cerise JE, Jahoda CAB, Christiano AM. Microenvironmental reprogramming by three-dimensional culture enables dermal papilla cells to induce de novo human hair-follicle growth. Proc Natl Acad Sci U S A. 2013;110: 19679–19688. doi: 10.1073/pnas.1309970110 24145441 10.1073/pnas.1309970110 PMC3856847

[17] Miao Y, Sun YB, Liu BC, Jiang JD, Hu ZQ. Controllable production of transplantable adult human high-passage dermal papilla spheroids using 3D matrigel culture. Tissue Eng Part A. 2014;20: 2329–2338. doi: 10.1089/ten.TEA.2013.0547 24528213 10.1089/ten.tea.2013.0547 PMC4161057

[18] Lin B, Miao Y, Wang J, Fan Z, Du L, Su Y, et al Surface tension guided hanging-drop: producing controllable 3D spheroid of high-passaged human dermal papilla cells and forming inductive microtissues for hair-follicle regeneration. ACS Appl Mater Interfaces. 2016;8: 5906–5916. doi: 10.1021/acsami.6b00202 26886167 10.1021/acsami.6b00202

[19] Thane K, Ingenito EP, Hoffman AM. Lung regeneration and translational implications of the postpneumonectomy model. Transl Res. 2014;163: 363–376. doi: 10.1016/j.trsl.2013.11.010 24316173 10.1016/j.trsl.2013.11.010

[20] Fiegel HC, Kaufmann PM, Bruns H, Kluth D, Horch RE, Vacanti JP, et al Hepatic tissue engineering: from transplantation to customized cell-based liver directed therapies from the laboratory. J Cell Mol Med. 2008;12: 56–66. doi: 10.1111/j.1582-4934.2007.00162.x 18021311 10.1111/j.1582-4934.2007.00162.x PMC3823472

[21] Sennett R, Rendl M. Mesenchymal-epithelial interactions during hair follicle morphogenesis and cycling. Semin Cell Dev Biol. 2012;23: 917–927. doi: 10.1016/j.semcdb.2012.08.011 22960356 10.1016/j.semcdb.2012.08.011 PMC3496047

[22] Kloepper JE, Baris OR, Reuter K, Kobayashi K, Weiland D, Vidali S, et al Mitochondrial function in murine skin epithelium is crucial for hair follicle morphogenesis and epithelial-mesenchymal interactions. J Invest Dermatol. 2015;135: 679–689. doi: 10.1038/jid.2014.475 25371971 10.1038/jid.2014.475

[23] Ohyama M, Veraitch O. Strategies to enhance epithelial-mesenchymal interactions for human hair follicle bioengineering. J Dermatol Sci. 2013;70: 78–87. doi: 10.1016/j.jdermsci.2013.02.004 23557720 10.1016/j.jdermsci.2013.02.004

[24] Hazeltine LB, Selekman JA, Palecek SP. Engineering the human pluripotent stem cell microenvironment to direct cell fate. Biotechnol Adv. 2013;31: 1002–1019. doi: 10.1016/j.biotechadv.2013.03.002 23510904 10.1016/j.biotechadv.2013.03.002 PMC3758782

[25] Huang C, Du Y, Nabzdyk CS, Ogawa R, Koyama T, Orgill D, et al Regeneration of hair and other skin appendages: A microenvironment-centric view. Wound Repair Regen. 2016;24: 759–766. doi: 10.1111/wrr.12451 27256925 10.1111/wrr.12451

[26] Kim SH, Turnbull J, Guimond S. Extracellular matrix and cell signalling: the dynamic cooperation of integrin, proteoglycan and growth factor receptor. J Endocrinol. 2011;209: 139–151. doi: 10.1530/JOE-10-0377 21307119 10.1530/JOE-10-0377

[27] Jinno H, Morozova O, Jones KL, Biernaskie JA, Paris M, Hosokawa R, et al Convergent genesis of an adult neural crest-like dermal stem cell from distinct developmental origins. Stem Cells. 2010;28: 2027–2040. doi: 10.1002/stem.525 20848654 10.1002/stem.525 PMC3087810

[28] Miao Y, Sun YB, Sun XJ, Du BJ, Jiang JD, Hu ZQ. Promotional effect of platelet-rich plasma on hair follicle reconstitution in vivo. Dermatol Surg. 2013;39: 1868–1876. doi: 10.1111/dsu.12292 24118627 10.1111/dsu.12292

[29] Schormann W, Hammersen FJ, Brulport M, Hermes M, Bauer A, Rudolph C, et al Tracking of human cells in mice[J]. Histochem Cell Biol. 2008;130: 329–338. doi: 10.1007/s00418-008-0428-5 18425526 10.1007/s00418-008-0428-5

[30] Zheng Y, Nace A, Chen W, Watkins K, Sergott L, Homan Y, et al Mature hair follicles generated from dissociated cells: a universal mechanism of folliculoneogenesis. Dev Dyn. 2010;239: 2619–2626. doi: 10.1002/dvdy.22398 21038446 10.1002/dvdy.22398

[31] Salmons B, Gunzburg WH. Therapeutic application of cell microencapsulation in cancer. Adv Exp Med Biol. 2010;670: 92–103. 20384221 10.1007/978-1-4419-5786-3_9

[32] Lohr M, Hoffmeyer A, Kroger J, Freund M, Hain J, Holle A, et al Microencapsulated cell-mediated treatment of inoperable pancreatic carcinoma. Lancet. 2001;57: 1591–1592. 10.1016/s0140-6736(00)04749-8 11377651

[33] Yen CM, Chan CC, Lin SJ. High-throughput reconstitution of epithelial-mesenchymal interaction in folliculoid microtissues by biomaterial-facilitated self-assembly of dissociated heterotypic adult cells. Biomaterials. 2010;31: 4341–4352. doi: 10.1016/j.biomaterials.2010.02.014 20206989 10.1016/j.biomaterials.2010.02.014

[34] Bruneau BG. Signaling and transcriptional networks in heart development and regeneration. Cold Spring Harb Perspect Biol. 2013;5(3):a008292 doi: 10.1101/cshperspect.a008292 23457256 10.1101/cshperspect.a008292 PMC3578359

[35] Wang X, Tredget EE, Wu Y. Dynamic signals for hair follicle development and regeneration. Stem Cells Dev. 2012;21(1):7–18. doi: 10.1089/scd.2011.0230 21787229 10.1089/scd.2011.0230

